# Cardiovascular magnetic resonance‐estimated pulmonary capillary wedge pressure, congestion markers, and effect of empagliflozin in patients with heart failure with reduced ejection fraction and dysglycaemia (SUGAR‐DM‐HF)

**DOI:** 10.1002/ejhf.3570

**Published:** 2025-01-10

**Authors:** Joanna Osmanska, Kieran F. Docherty, Colin Berry, Pardeep S. Jhund, Kenneth Mangion, Patrick B. Mark, John J.V. McMurray, Giles Roditi, Naveed Sattar, Mark C. Petrie, Ross T. Campbell, Matthew M.Y. Lee

**Affiliations:** ^1^ School of Cardiovascular and Metabolic Health, British Heart Foundation Glasgow Cardiovascular Research Centre, University of Glasgow Glasgow UK

## Introduction

Accurately estimating left ventricular (LV) filling pressure is crucial for guiding effective treatment decisions. Invasively measured pulmonary capillary wedge pressure (PCWP) is the gold standard method of indirectly estimating LV filling pressure. However, the routine invasive measurement of PCWP is limited by the availability of right heart catheterization and the risk of procedure‐related complications. Cardiovascular magnetic resonance (CMR) imaging is the gold standard method to assess LV geometry and function. A CMR imaging‐based model to non‐invasively estimate PCWP (CMR‐PCWP) was recently developed and correlated well with invasively measured PCWP, and was an independent predictor of the risk of mortality after adjustment for LV volumes in patients with suspected heart failure (HF).^1^ Additionally, in an imaging substudy of the UK Biobank involving 39 163 participants, raised CMR‐PCWP was identified as an independent risk factor for incident HF and major adverse cardiovascular events at the population level.^2^ The associations with this non‐invasive surrogate of LV filling pressure and other established imaging‐based and circulating biomarkers of congestion have not been described.

In this exploratory analysis of the Studies of Empagliflozin and its Cardiovascular, Renal and Metabolic Effects in Patients with Diabetes Mellitus, or Pre‐diabetes, and Heart Failure trial (SUGAR‐DM‐HF, NCT03485092), we assessed the association between CMR‐PCWP and other imaging and circulating biomarker measures of congestion in patients with HF and reduced ejection fraction (HFrEF) and type 2 diabetes or pre‐diabetes.^3^ Additionally, we investigated the effect of the sodium–glucose cotransporter 2 (SGLT2) inhibitor empagliflozin on CMR‐PCWP and its relationship with the cardiac remodelling effect of empagliflozin. Our hypothesis was that CMR‐PCWP could serve as a non‐invasive marker of LV filling pressures in HFrEF and may be modulated by empagliflozin.

## Methods

SUGAR‐DM‐HF was a multicentre, randomized, double‐blind, placebo‐controlled trial. The study design and main results are published.^3^ A total of 105 patients in New York Heart Association (NYHA) functional class II–III, with a LV ejection fraction (LVEF) ≤40%, and type 2 diabetes, or pre‐diabetes (glycated haemoglobin 39–47 mmol/mol [5.7–6.4%]), and without persistent/permanent atrial fibrillation/flutter, were randomly assigned 1:1 to empagliflozin 10 mg daily or placebo in addition to standard HFrEF pharmacological therapy. The randomization sequence was performed in blocks of 4 and stratified by (1) age (<65 years, ≥65 years) and (2) diabetes or pre‐diabetes status. Transthoracic echocardiography (TTE) was performed at screening (0–12 weeks before baseline). Physical examination, lung ultrasound (LUS), and CMR (MAGNETOM Prisma 3 T scanner, Siemens) were performed at baseline and 36 weeks along with venous blood collection for measurement of circulating biomarkers of congestion. CMR analysis was performed by a single observer (M.M.Y.L.), who was blinded to subject ID, scan date and time, clinical data, and randomization arm. CMR‐PCWP (mmHg) was calculated: 6.1352 + (0.07204 × left atrial volume) + (0.02256 × LV mass).^1^ A modified version of the ADVOR congestion score (adapted from the Acetazolamide in Decompensated Heart Failure with Volume Overload [ADVOR] trial^4^ and the Dapagliflozin versus Metolazone in Heart Failure Resistant to Loop Diuretics [DAPA‐RESIST] trial^5^) was calculated. The original ADVOR congestion score ranges from 0 to 10, summing scores for oedema (0 to 4), pleural effusion (0 to 3), and ascites (0 to 3), with higher scores indicating greater congestion.^4^ The modified ADVOR score, also ranging from 0 to 10, uses LUS to assess pleural effusion instead of standard physical examination, providing a more objective measure of its presence and severity.^5^ Analyses were performed using Stata version 17 (StataCorp., College Station, TX, USA). Log‐transformations were applied to N‐terminal pro‐B‐type natriuretic peptide (NT‐proBNP), growth differentiation factor‐15, and carbohydrate antigen 125 to meet model distributional assumptions. No adjustments were made for multiple comparisons.

## Results

Of 105 patients with HFrEF randomized, mean age was 69 years, 73% were male, mean LVEF 33% and median NT‐proBNP was 466 (interquartile range [IQR] 177‐1120) pg/ml (*Table* *1*). Most baseline characteristics were well balanced between the treatment groups.^3^ Baseline CMR‐PCWP was calculable in all 105 patients, and paired baseline and 36‐week CMR data were available in 95 patients (45 and 50 randomized to empagliflozin and placebo, respectively).

**Table 1:** Baseline characteristics and laboratory values by baseline cardiovascular magnetic resonance‐estimated pulmonary capillary wedge pressure categories

	All (*n* = 105)	PCWP <15 mmHg (*n* = 53)	PCWP ≥15 mmHg (*n* = 52)	*p* value
Age (years)	68.7 ± 11.1	68.9 ± 11.1	68.5 ± 11.2	0.86
Male sex	77 (73)	32 (60)	45 (87)	**<0.01**
Body mass index (kg/m^2^)	30.7 ± 5.5	29.4 ± 5.6	31.9 ± 5.1	**0.02**
Heart rate (bpm)	66.4 ± 11.5	67.8 ± 11.6	65.0 ± 11.3	0.21
Systolic blood pressure (mmHg)	128.0 ± 20.0	125.0 ± 17.4	131.1 ± 22.1	0.12
Heart failure status				
Duration of heart failure (years)	2.1 [1.0–4.8]	2.2 [1.0–4.3]	1.9 [1.0–4.8]	0.94
Prior heart failure hospitalization	52 (50)	27 (51)	25 (48)	0.77
New York Heart Association class II	81 (77)	37 (70)	44 (85)	0.07
6‐min walk distance (m)^a^	344.5 ± 110.8	334.6 ± 122.5	354.3 ± 98.4	0.39
KCCQ‐CSS	61.4 ± 23.2	57.5 ± 24.0	65.4 ± 21.9	0.08
Other medical history				
Coronary artery disease	74 (70)	34 (64)	40 (77)	0.15
Previous myocardial infarction	62 (59)	29 (55)	33 (63)	0.36
Previous percutaneous coronary intervention	42 (40)	20 (38)	22 (42)	0.63
Previous coronary artery bypass graft	18 (17)	6 (11)	12 (23)	0.11
Hypertension	74 (70)	38 (72)	36 (69)	0.78
Stroke	11 (10)	8 (15)	3 (6)	0.12
Type 2 diabetes	82 (78)	42 (79)	40 (77)	0.77
Laboratory results				
Sodium (mmol/L)	138.3 ± 2.9	138.1 ± 2.9	138.4 ± 2.9	0.61
Urea (mmol/L)	8.0 ± 3.5	8.2 ± 3.6	7.8 ± 3.4	0.52
Albumin (g/L)	37.2 ± 2.8	37.3 ± 3.1	37.2 ± 2.5	0.87
Creatinine (μmol/L)	100.2 ± 31.8	100.6 ± 32.5	99.8 ± 31.5	0.90
eGFR (CKD‐EPI) (ml/min/1.73 m^2^)	67.3 ± 22.0	65.0 ± 21.5	69.6 ± 22.5	0.29
NT‐proBNP (pg/ml)	466 [177–1120]	354 [127–683]	611 [279–1454]	**0.01**
GDF‐15 (pg/ml)	2331 [1469–3390]	2301 [1630–3390]	2352 [1330–3466]	0.77
CA‐125 (U/ml)	11.2 [8.9–17.6]	11.3 [9.2–16.4]	10.9 [7.8–18.7]	0.77
Physical examination and ultrasound markers of congestion				
Elevated jugular venous pressure	3 (3)	0 (0)	3 (6)	0.08
Pulmonary crepitations	13 (12)	6 (11)	7 (13)	0.74
Peripheral oedema	67 (64)	30 (57)	37 (71)	0.12
Ankle	65 (97)	29 (97)	36 (97)	0.22
Knee	1 (1)	0 (0)	1 (3)	
Thigh	1 (1)	1 (3)	0 (0)	
Pleural effusion on lung ultrasound	3 (3)	0 (0)	3 (6)	0.08
Total number of B‐lines^b^	5 [2–9]	3 [1–7]	6 [4–11]	**<0.01**
Estimated pulmonary artery systolic pressure (mmHg)^c^	17.5 ± 10.0	15.2 ± 7.5	19.7 ± 11.7	**0.03**
Estimated right atrial pressure (mmHg)^d^	3.2 ± 2.4	2.6 ± 0.7	3.9 ± 3.2	**<0.01**
Average E/e'^e^	11.7 ± 4.9	10.9 ± 4.2	12.6 ± 5.5	0.08
Modified ADVOR score	2 [0–2]	2 [0–2]	2 [0–2]	0.07
Medication				
ACEi/ARB/ARNi	100 (95)	51 (96)	49 (94)	0.63
ACEi/ARB	64 (61)	32 (60)	32 (62)	0.90
ARNi	36 (34)	19 (36)	17 (33)	0.73
Beta‐blocker	96 (91)	46 (87)	50 (96)	0.09
Mineralocorticoid receptor antagonist	63 (60)	30 (57)	33 (63)	0.47
Loop diuretic	60 (57)	31 (58)	29 (56)	0.78
Dose of loop diuretic (mg)	40 [40–80]	40 [40–80]	40 [40–80]	0.22
Cardiovascular magnetic resonance‐derived measurements				
PCWP (mmHg)	15.1 ± 2.5	13.2 ± 1.3	17.0 ± 1.8	**<0.001**
Left ventricular ejection fraction (%)	32.5 ± 9.8	34.3 ± 10.1	30.7 ± 9.1	0.05
Left ventricular end‐systolic volume (ml)	152.5 ± 62.0	126.5 ± 54.7	179.0 ± 58.0	**<0.001**
Left ventricular end‐diastolic volume (ml)	220.9 ± 67.2	187.6 ± 56.3	254.8 ± 60.5	**<0.001**
Left ventricular stroke volume (ml)	68.4 ± 20.9	61.2 ± 17.6	75.8 ± 21.6	**<0.001**
Left ventricular mass (g)	128.0 ± 43.6	108.4 ± 32.4	148.1 ± 44.6	**<0.001**

Data are presented as mean ± standard deviation, *n* (%), or median [interquartile range].

ACEi, angiotensin‐converting enzyme inhibitor; ADVOR, Acetazolamide in Decompensated Heart Failure with Volume Overload; ARB, angiotensin receptor blocker; ARNi, angiotensin receptor–neprilysin inhibitor; CA‐125, carbohydrate antigen 125; CKD‐EPI, Chronic Kidney Disease Epidemiology Collaboration; eGFR, estimated glomerular filtration rate; GDF‐15, growth differentiation factor‐15; KCCQ‐CSS, Kansas City Cardiomyopathy Questionnaire clinical summary score; NT‐proBNP, N‐terminal pro‐B‐type natriuretic peptide; PCWP, pulmonary capillary wedge pressure.

^a^ *n* = 93 (*n* = 46 empagliflozin group; *n* = 47 placebo group).

^b^ *n* = 104 (excluding one patient from the placebo group with pulmonary fibrosis); number of B‐lines over 8 zones.

^c^ *n* = 93 (*n* = 45 empagliflozin group; *n* = 48 placebo group).

^d^ *n* = 97 (n = 46 empagliflozin group; *n* = 51 placebo group).

^e^ *n* = 101 (*n* = 49 empagliflozin group; *n* = 52 placebo group) (excluding three patients with prior valve interventions: two with tissue aortic valve replacement [*n* = 1 empagliflozin group; *n* = 1 placebo group] and one with mitral valve repair [*n* = 1 empagliflozin group]).

### Baseline characteristics according to cardiovascular magnetic resonance‐estimated pulmonary capillary wedge pressure

Mean baseline CMR‐PCWP was 15.1 (SD 2.5) mmHg, and 52 (50%) patients had a baseline CMR‐PCWP ≥15 mmHg. Compared with a CMR‐PCWP <15 mmHg, patients with CMR‐PCWP ≥15 mmHg were more frequently male, had higher mean body mass index and higher concentrations of NT‐proBNP (*Table* *1*). There were no significant between‐group differences in background HF therapy use, including median loop diuretic dose.

### Association between baseline cardiovascular magnetic resonance‐estimated pulmonary capillary wedge pressure and circulating and imaging‐based measures of congestion

**Figure 1:**
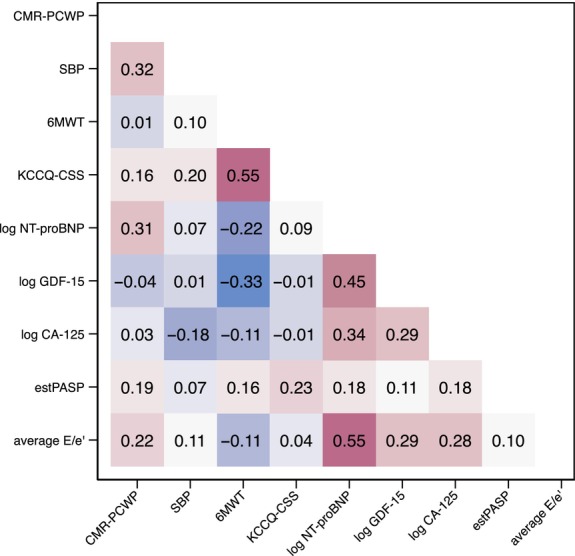
Correlation between baseline cardiovascular magnetic resonance‐estimated pulmonary capillary wedge pressure (CMR‐PCWP) and baseline characteristics including circulating and ultrasound‐based measures. Data are presented as correlation heatmap with Pearson correlation coefficients. 6MWT, 6‐min walk test; CA‐125, carbohydrate antigen 125; estPASP, estimated pulmonary artery systolic pressure; GDF‐15, growth differentiation factor‐15; KCCQ‐CSS, Kansas City Cardiomyopathy Questionnaire clinical summary score; NT‐proBNP, N‐terminal pro‐B‐type natriuretic peptide; SBP, systolic blood pressure.

Compared with a CMR‐PCWP <15 mmHg, patients with CMR‐PCWP ≥15 mmHg had a greater total number of B‐lines on LUS and higher TTE‐estimated pulmonary artery systolic pressure and right atrial pressure (*Table* *1*). There were no significant between‐group differences in clinical examination findings of congestion or the modified ADVOR congestion score. CMR‐PCWP moderately correlated with systolic blood pressure (Pearson's *r* = 0.32, *p* = 0.0011), log NT‐proBNP (*r* = 0.31, *p* = 0.0012), and total number of B‐lines on LUS (Spearman's rho = 0.34, *p* < 0.001) (*Figure* *1*). CMR‐PCWP weakly correlated with TTE estimates of LV filling pressure, with average E/e' *r* = 0.22, *p* = 0.03.

### Association between baseline cardiovascular magnetic resonance‐estimated pulmonary capillary wedge pressure and cardiovascular magnetic resonance‐measured cardiac volumes and function

**Figure 2:**
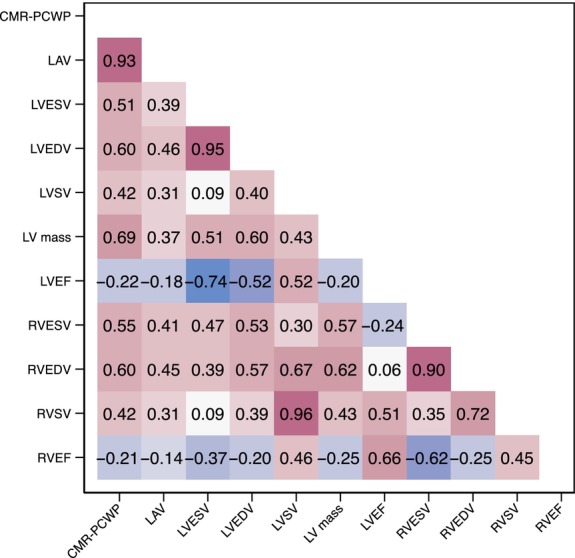
Correlation between baseline cardiovascular magnetic resonance‐estimated pulmonary capillary wedge pressure (CMR‐PCWP) and baseline CMR‐derived measurements. Data are presented as correlation heatmap with Pearson correlation coefficients. LAV, left atrial volume; LV, left ventricular; LVEDV, left ventricular end‐diastolic volume; LVEF, left ventricular ejection fraction; LVESV, left ventricular end‐systolic volume; LVSV, left ventricular stroke volume; RVEDV, right ventricular end‐diastolic volume; RVEF, right ventricular ejection fraction; RVESV, right ventricular end‐systolic volume; RVSV, right ventricular stroke volume.

Cardiovascular magnetic resonance‐estimated PCWP positively correlated with CMR‐measured volumes (LV end‐systolic volume: *r* = 0.51, *p* < 0.001; LV end‐diastolic volume: *r* = 0.60, *p* < 0.001; right ventricular [RV] end‐systolic volume: *r* = 0.55, *p* < 0.001; RV end‐diastolic volume: *r* = 0.60, *p* < 0.001) (*Figure* *2*). There were weak inverse correlations between CMR‐PCWP and CMR‐derived LVEF (*r* = −0.22, *p* < 0.001) and CMR‐derived RV ejection fraction (*r* = −0.21, *p* < 0.001).

### Relationship between the effect of empagliflozin and cardiovascular magnetic resonance‐estimated pulmonary capillary wedge pressure

**Figure 3:**
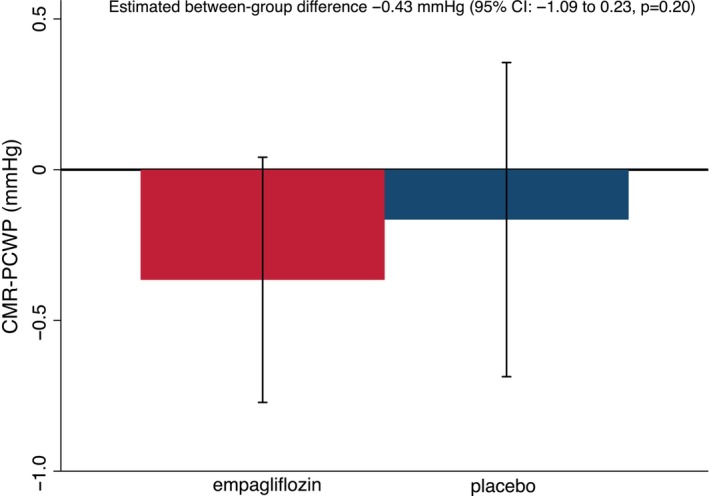
Change in cardiovascular magnetic resonance‐estimated pulmonary capillary wedge pressure (CMR‐PCWP) from baseline to 36 weeks. CI, confidence interval.

Mean CMR‐PCWP decreased from baseline to 36 weeks by −0.37 (95% confidence interval −0.78 to 0.05) mmHg and −0.17 (−0.69 to 0.36) mmHg in the empagliflozin and placebo groups, respectively; placebo‐corrected difference −0.43 mmHg (−1.09 to 0.23, *p* = 0.20) (*Figure* *3*). The beneficial effect of empagliflozin on reducing LV end‐systolic volume index was not modified by baseline CMR‐PCWP (*p* interaction = 0.98).

## Discussion

In this exploratory analysis of the SUGAR‐DM‐HF trial, a novel non‐invasive CMR‐based method of estimating PCWP was associated with other established circulating (NT‐proBNP) and imaging‐based (average E/e') surrogates of LV filling pressure along with the degree of pulmonary congestion (LUS‐measured B‐lines). Higher estimated CMR‐PCWP was correlated with greater adverse cardiac remodelling, as measured by CMR. Clinically, these correlations suggest that CMR‐PCWP could facilitate early detection of worsening HF and guide targeted interventions (such as increasing diuretic doses or commencing diuretics). However, further research in larger, well‐powered studies is needed to validate these findings and assess their impact on patient management. To our knowledge, this is the first randomized controlled trial to utilize CMR‐PCWP.

Cardiovascular magnetic resonance imaging is recommended in guidelines to provide information regarding aetiology in patients with HFrEF. The novel CMR‐based estimate described by Garg *et al*.^1^ used in this analysis offers an easily obtainable estimate of LV filling pressure derived from standard measurements (left atrial volume and LV mass) which could be routinely added to CMR reports. Our finding of significant, albeit moderate, correlations between CMR‐PCWP and NT‐proBNP, and LUS‐measured B‐lines, adds to the potential value of this new surrogate measure in assessing patients with HFrEF undergoing CMR to identify those with elevated LV filling pressure who may benefit from further assessment as to the presence of congestion potentially requiring treatment intensification (e.g. diuretics). There was no significant association between an elevated CMR‐PCWP and clinical findings of systemic venous congestion (e.g. peripheral oedema, jugular venous distension, modified ADVOR score). However, this may reflect the limitations of clinical examination‐based assessment of congestion and its relatively low prevalence in this trial. Moreover, in a larger cohort of 454 patients with HF, an elevated CMR‐PCWP was significantly associated with peripheral oedema.^6^

Although CMR‐PCWP and NT‐proBNP were correlated in SUGAR‐DM‐HF, empagliflozin significantly reduced NT‐proBNP but did not affect CMR‐PCWP. Possible reasons for this discrepancy include the modest correlation between the measures, insufficient power to detect changes in CMR‐PCWP, and the fact that baseline CMR‐PCWP was not elevated in many patients. Despite empagliflozin's effects on NT‐proBNP, its proposed mechanisms, such as preload reduction, may not have sufficiently altered LV filling pressures within the study's 36‐week timeframe. Additionally, early changes in PCWP, such as those observed at 12 weeks in the Empire HF (Empagliflozin in heart failure patients with reduced ejection fraction) trial, may have diminished by the 36‐week mark.^7^

In both the original derivation cohort and a recent external validation cohort, CMR‐PCWP predicted mortality and the risk of HF hospitalization, adding to its potential value as a routine CMR‐based measure to risk stratify patients.^1^, ^6^ Due to the limited size of the SUGAR‐DM‐HF trial and relatively short follow‐up time, we were unable to explore the relationship between CMR‐PCWP and outcomes.

As well as its potential role in assessing patients with HFrEF, CMR‐PCWP may have a role in assessing patients with suspected HF with preserved ejection fraction (HFpEF). In the original derivation cohort, CMR‐PCWP significantly outperformed TTE‐based estimated LV filling pressure in identifying patients with elevated invasively measured PCWP.^1^ TTE‐derived estimates of LV filling pressures are recommended in the assessment of patients with suspected HFpEF and invasive testing is suggested in those with equivocal echocardiography but a high clinical suspicion of HFpEF.^8^, ^9^, ^10^ The apparent superior performance of CMR‐PCWP over TTE‐based estimates raises the possibility that CMR‐PCWP may be a useful tool for assessing patients with suspected HFpEF in whom an estimate of LV filling pressure can be made without the need for invasive right heart catheterization to confirm or refute the diagnosis. Furthermore, in the HFpEF‐Stress trial, which recruited 75 patients with exertional dyspnoea and diastolic dysfunction, non‐invasive CMR‐PCWP correlated well with the invasive reference both at rest and during exercise stress.^11^

We did not observe a significant reduction in CMR‐PCWP in patients randomized to empagliflozin as compared with placebo. Similarly, in the placebo‐controlled Empire HF, there was no significant reduction in invasively measured PCWP at rest in patients with HFrEF randomized to empagliflozin for 12 weeks.^7^ In both trials there were similar favourable effects on reverse cardiac remodelling, and there was a significant 28% reduction in NT‐proBNP in SUGAR‐DM‐HF that was not seen in Empire HF.^12^ Conversely, in the EMBRACE‐HF (Empagliflozin Evaluation by Measuring Impact on Hemodynamics in Patients with Heart Failure) trial, empagliflozin reduced mean pulmonary artery diastolic pressure measured with the CardioMEMs implantable pressure monitor, an effect seen as early as 1 week following treatment initiation.^13^ This effect was not accompanied by any significant change in NT‐proBNP suggesting that any effect of empagliflozin on reducing pulmonary pressures may be secondary to a direct effect on the pulmonary vasculature (e.g. mediated by a reduction in endothelin‐1) rather than being secondary to a substantial reduction in left‐sided filling pressures. Taken together, these data along with other studies showing a modest effect of SGLT2 inhibitors on reducing NT‐proBNP and other circulating biomarkers associated with congestion, as well as a minimal long‐term diuretic effect, suggest that decongestion is not a predominant mechanism underlying the clinical benefits of SGLT2 inhibitors in HF.^14^, ^15^, ^16^, ^17^, ^18^

This *post‐hoc* analysis has several limitations. The analyses were not pre‐specified. The trial was not powered to detect the effect of empagliflozin treatment on CMR‐PCWP, with the modest sample size (*n* = 105) likely limiting the statistical power of this *post‐hoc* analysis. Due to the small sample size and the *post‐hoc* nature of this analysis, additional subgroup analyses were not performed. Additionally, most TTEs were performed on a different day from the baseline CMR (median difference 7 days [IQR 3–12]). LV filling pressure is dynamic and can increase with exercise, which we were unable to assess as all scans were performed at rest. The follow‐up period was relatively short (36 weeks). Potential confounding variables include differences in baseline characteristics.

## Conclusion

In the SUGAR‐DM‐HF trial, non‐invasive CMR‐PCWP was significantly associated with other established circulating and echocardiographic biomarkers of LV filling pressure and LUS‐assessed pulmonary congestion. Potential clinical uses of CMR‐PCWP include: (1) enabling a non‐invasive estimate of filling pressure as an alternative to echocardiography; (2) confirming HF diagnosis by detecting elevated PCWP during CMR; (3) prompting further investigation in patients without known HF, such as those with cardiomyopathy, where elevated CMR‐PCWP may suggest reclassification to HF; and (4) opportunistically guiding diuretic adjustment in patients with elevated PCWP detected during CMR. There was no significant effect of empagliflozin on reducing CMR‐PCWP. Future research should focus on longer‐term studies, diverse patient populations, and the impact of different interventions on CMR‐PCWP and related biomarkers.

### Funding

This trial was supported by an investigator‐initiated study grant from Boehringer Ingelheim. Boehringer Ingelheim has provided support in terms of funding and investigational medicinal product (IMP). The funder had no role in the design and conduct of the study; collection, management, analysis, and interpretation of the data; preparation or decision to submit the article for publication. Drs. Berry, McMurray, Sattar, and Petrie are supported by a British Heart Foundation Centre of Research Excellence Grant RE/18/6/34217.

**Conflict of interest**: K.F.D. reports that his employer, the University of Glasgow, has been renumerated by AstraZeneca for work relating to clinical trials. He has received speaker's honoraria from AstraZeneca, Pharmacosmos, Boehringer Ingelheim, Translational Medicine Academy and Radcliffe Cardiology, has served on an advisory board for Us2.ai and holds equity in the company, has served on an advisory board and served on a clinical endpoint committee for Bayer AG, and has received grant support from Boehringer Ingelheim, Roche Diagnostics and AstraZeneca (paid to his institution). C.B. is employed by the University of Glasgow which holds consultancy and research agreements for his work with Abbott Vascular, AstraZeneca, Boehringer Ingelheim, CorFlow, Coroventis, HeartFlow, Menarini, MSD, Novartis, Servier, Siemens Healthcare, TherOx and Valo Health. P.S.J. reports speaker's fees from AstraZeneca, Novartis, Alkem Metabolics, ProAdWise Communications, Sun Pharmaceuticals; advisory board fees from AstraZeneca, Boehringer Ingelheim, Novartis; research funding from AstraZeneca, Boehringer Ingelheim, Analog Devices Inc. P.S.J.'s employer the University of Glasgow has been remunerated for clinical trial work from AstraZeneca, Bayer AG, Novartis and NovoNordisk. Director, Global Clinical Trial Partners (GCTP). P.B.M. reports consultancy or speaker fees from Boehringer Ingelheim, AstraZeneca, Pharmacosmos, GSK, Bayer, and Vifor Fresenius Medical Care Renal Pharma. J.J.V.M. has received payments through Glasgow University from work on clinical trials, consulting and other activities from Alnylam, Amgen, AstraZeneca, Bayer, Boehringer Ingelheim, BMS, Cardurion, Cytokinetics, Dal‐Cor, GSK, Ionis, KBP Biosciences, Novartis, Pfizer, Theracos; personal lecture fees from the Corpus, Abbott, Hikma, Sun Pharmaceuticals, Medscape/Heart.Org, Radcliffe Cardiology, Servier, Director, Global Clinical Trial Partners (GCTP). G.R. reports speaker honoraria from GE Healthcare and Bracco SpA and a non‐remunerated consultancy for Canon Medical. N.S. has consulted for and/or received speaker honoraria from Abbott Laboratories, AbbVie, Amgen, AstraZeneca, Boehringer Ingelheim, Eli Lilly, Hanmi Pharmaceuticals, Janssen, Menarini‐Ricerche, Novartis, Novo Nordisk, Pfizer, Roche Diagnostics, and Sanofi; he has received grant support paid to his university from AstraZeneca, Boehringer Ingelheim, Novartis, and Roche Diagnostics outside the submitted work. M.C.P. has received research funding from Boehringer Ingelheim, Roche, SQ Innovation Inc, AstraZeneca, Novartis, Novo Nordisk, Medtronic, Boston Scientific, Pharmacosmos; consultancy and trial committees: Akero, Applied Therapeutics, Amgen, AnaCardio, Biosensors, Boehringer Ingelheim, Corteria, Novartis, AstraZeneca, Novo Nordisk, Abbvie, Bayer, Horizon Therapeutics, Foundry, Takeda, Cardiorentis, Pharmacosmos, Siemens, Eli Lilly, Vifor, New Amsterdam, Moderna, Teikoku, LIB Therapeutics, 3R Lifesciences, Reprieve, FIRE 1; Director of Global Clinical Trial Partners (GCTP). R.T.C. has received research funding from Boehringer Ingelheim, Roche, SQ Innovation Inc, and AstraZeneca; consultancy fees from Boehringer Ingelheim and speaker fees from AstraZeneca. M.M.Y.L. has received grant support from AstraZeneca, Boehringer Ingelheim, and Roche Diagnostics; he serves on clinical endpoint committees for Bayer and GSK, and steering committees for Cytokinetics. All other authors have nothing to disclose.

## References

[1] Garg P , Gosling R , Swoboda P , Jones R , Rothman A , Wild JM , *et al*. Cardiac magnetic resonance identifies raised left ventricular filling pressure: Prognostic implications. Eur Heart J 2022;43:2511–2522. 10.1093/eurheartj/ehac207 35512290 PMC9259376

[2] Thomson RJ , Grafton‐Clarke C , Matthews G , Swoboda PP , Swift AJ , Frangi A , *et al*. Risk factors for raised left ventricular filling pressure by cardiovascular magnetic resonance: Prognostic insights. ESC Heart Fail 2024;11:4148–4159. 10.1002/ehf2.15011 39132877 PMC11631267

[3] Lee MMY , Brooksbank KJM , Wetherall K , Mangion K , Roditi G , Campbell RT , *et al*. Effect of empagliflozin on left ventricular volumes in patients with type 2 diabetes, or prediabetes, and heart failure with reduced ejection fraction (SUGAR‐DM‐HF). Circulation 2021;143:516–525. 10.1161/CIRCULATIONAHA.120.052186 33186500 PMC7864599

[4] Mullens W , Dauw J , Martens P , Meekers E , Nijst P , Verbrugge FH , *et al*. Acetazolamide in Decompensated Heart Failure with Volume Overload trial (ADVOR): Baseline characteristics. Eur J Heart Fail 2022;24:1601–1610. 10.1002/ejhf.2587 35733283

[5] Ern Yeoh S , Osmanska J , Petrie MC , Brooksbank KJM , Clark AL , Docherty KF , *et al*. Dapagliflozin vs. metolazone in heart failure resistant to loop diuretics. Eur Heart J 2023;44:2966–2977. 10.1093/eurheartj/ehad341 37210742 PMC10424881

[6] Grafton‐Clarke C , Garg P , Swift AJ , Alabed S , Thomson R , Aung N , *et al*. Cardiac magnetic resonance left ventricular filling pressure is linked to symptoms, signs and prognosis in heart failure. ESC Heart Fail 2023;10:3067–3076. 10.1002/ehf2.14499 37596895 PMC10567675

[7] Omar M , Jensen J , Frederiksen PH , Kistorp C , Videbæk L , Poulsen MK , *et al*. Effect of empagliflozin on hemodynamics in patients with heart failure and reduced ejection fraction. J Am Coll Cardiol 2020;76:2740–2751. 10.1016/j.jacc.2020.10.005 33272368

[8] Andersen OS , Smiseth OA , Dokainish H , Abudiab MM , Schutt RC , Kumar A , *et al*. Estimating left ventricular filling pressure by echocardiography. J Am Coll Cardiol 2017;69:1937–1948. 10.1016/j.jacc.2017.01.058 28408024

[9] McDonagh TA , Metra M , Adamo M , Gardner RS , Baumbach A , Böhm M , *et al*. 2021 ESC Guidelines for the diagnosis and treatment of acute and chronic heart failure: Developed by the Task Force for the diagnosis and treatment of acute and chronic heart failure of the European Society of Cardiology (ESC). With the special contribution of the Heart Failure Association (HFA) of the ESC. Eur J Heart Fail 2022;24:4–131. 10.1002/ejhf.2333 35083827

[10] Pieske B , Tschöpe C , de Boer RA , Fraser AG , Anker SD , Donal E , *et al*. How to diagnose heart failure with preserved ejection fraction: The HFA‐PEFF diagnostic algorithm: A consensus recommendation from the Heart Failure Association (HFA) of the European Society of Cardiology (ESC). Eur Heart J 2019;40:3297–3317. 10.1093/eurheartj/ehz641 31504452

[11] Backhaus SJ , Schulz A , Lange T , Evertz R , Kowallick JT , Hasenfuß G , *et al*. Rest and exercise‐stress estimated pulmonary capillary wedge pressure using real‐time free‐breathing cardiovascular magnetic resonance imaging. J Cardiovasc Magn Reson 2024;26:101032. 10.1016/j.jocmr.2024.101032 38431079 PMC10950869

[12] Jensen J , Omar M , Kistorp C , Poulsen MK , Tuxen C , Gustafsson I , *et al*. Twelve weeks of treatment with empagliflozin in patients with heart failure and reduced ejection fraction: A double‐blinded, randomized, and placebo‐ controlled trial. Am Heart J 2020;228:47–56. 10.1016/j.ahj.2020.07.011 32798787

[13] Nassif ME , Qintar M , Windsor SL , Jermyn R , Shavelle DM , Tang F , *et al*. Empagliflozin effects on pulmonary artery pressure in patients with heart failure: Results from the EMBRACE‐HF trial. Circulation 2021;143:1673–1686. 10.1161/CIRCULATIONAHA.120.052503 33550815

[14] Yeoh SE , Docherty KF , Campbell RT , Jhund PS , Hammarstedt A , Heerspink HJL , *et al*. Endothelin‐1, outcomes in patients with heart failure and reduced ejection fraction, and effects of dapagliflozin: Findings from DAPA‐HF. Circulation 2023;147:1670–1683. 10.1161/CIRCULATIONAHA.122.063327 37039015 PMC10212584

[15] Docherty KF , McDowell K , Welsh P , Osmanska J , Anand I , de Boer RA , *et al*. Association of carbohydrate antigen 125 on the response to dapagliflozin in patients with heart failure. J Am Coll Cardiol 2023;82:142–157. 10.1016/j.jacc.2023.05.011 37407113

[16] Schork A , Saynisch J , Vosseler A , Jaghutriz BA , Heyne N , Peter A , *et al*. Effect of SGLT2 inhibitors on body composition, fluid status and renin‐angiotensin‐aldosterone system in type 2 diabetes: A prospective study using bioimpedance spectroscopy. Cardiovasc Diabetol 2019;18:46. 10.1186/s12933-019-0852-y 30953516 PMC6451223

[17] Scholtes RA , Muskiet MHA , van Baar MJB , Hesp AC , Greasley PJ , Karlsson C , *et al*. Natriuretic effect of two weeks of dapagliflozin treatment in patients with type 2 diabetes and preserved kidney function during standardized sodium intake: Results of the DAPASALT trial. Diabetes Care 2021;44:440–447. 10.2337/dc20-2604 33318125 PMC7818331

[18] Mordi NA , Mordi IR , Singh JS , McCrimmon RJ , Struthers AD , Lang CC . Renal and cardiovascular effects of SGLT2 inhibition in combination with loop diuretics in patients with type 2 diabetes and chronic heart failure: The RECEDE‐CHF trial. Circulation 2020;142:1713–1724. 10.1161/CIRCULATIONAHA.120.048739 32865004 PMC7594536

